# Reply to: Genomic insights into the 2020 mass die-off event among African elephants

**DOI:** 10.1038/s41467-025-63447-6

**Published:** 2025-09-26

**Authors:** Laura E. Rosen, Arnoud H. M. van Vliet, Chris M. Foggin

**Affiliations:** 1 Victoria Falls Wildlife Trust, Victoria Falls, Zimbabwe; 2 Department of Comparative Biomedical Sciences, School of Veterinary Medicine, Faculty of Health and Medical Sciences, University of Surrey, Guildford, UK

**Keywords:** Clinical microbiology, Bacterial infection

**replying to** A. Rasmussen et al. *Nature Communications* 10.1038/s41467-025-63446-7 (2025)

We thank Rasmussen et al. for their interest in our paper^1^, where we isolated the unnamed *Pasteurella* sp. currently known as Bisgaard taxon 45 in association with elephant mortalities in Zimbabwe in 2020. Rasmussen et al. furthered the investigation into the strain of Bisgaard taxon 45 from our study and compared it to other isolates of this organism. The manuscript by Rasmussen et al. provides compelling new evidence of virulence factors unique to the strain isolated from this mortality event. The fact that the isolate from our study contained genes absent from other Bisgaard taxon 45 isolates is of great interest to us, as is the reported similarity to proteins produced by *Neisseria meningitidis*, given that isolate from the 2020 mortality event appeared to have an affinity for the brain based on histological samples and at least some of the mortalities appeared peracute.

We acknowledge that pmHAS is a hyaluronan synthase^2^ rather than a hyaluronidase as in the reference cited in our study^3^. However, we are encouraged that the findings from the study by Rasmussen et al. otherwise build upon our results and lend further support to the potential for this organism to cause the mortality event we observed. We thank Rasmussen et al. for this valuable complementary study to better understand this potentially hypervirulent strain of Bisgaard taxon 45. Their work on potential virulence factors in Bisgaard taxon 45 provides a basis on which we can evaluate future isolates and better understand and diagnose this pathogen which had not previously been recognized in our region.

## Reporting Summary

Further information on research design is available in the Nature Portfolio Reporting Summary linked to this article.

## Supplementary information

Reporting Summary
